# 1-[3-(4-Nitro­phen­yl)propano­yl]urea acetic acid monosolvate

**DOI:** 10.1107/S1600536811039729

**Published:** 2011-10-08

**Authors:** Soraya Merzouki, Chabane Mouats, El-Eulmi Bendeif, Sebastien Pillet, Karim Bouchouit

**Affiliations:** a Laboratoire de Chimie Moléculaire, du Contrôle de l’Environnement et des Mesures Physico-Chimiques, Faculté des Sciences Exats, Département de Chimie, Université Mentouri de Constantine, 25000 Constantine, Algeria; b Laboratoire de Cristallographie, Résonance Magnétique et Modélisations, (CRM2, UMR CNRS 7036), Institut Jean Barriol, Nancy Université, BP 70239, Boulevard des Aiguillettes, 54506 Vandoeuvre-lès Nancy, France; c Département de Chimie, Faculté des Sciences, Université de Jijel, 18000-Jijel, Algeria

## Abstract

The title compound, C_10_H_11_N_3_O_4_·C_2_H_4_O_2_, was prepared by an electrochemical technique. In the crystal, acetic acid mol­ecules are involved in hydrogen bonding to two separate propano­ylurea mol­ecules, acting as a donor in an O—H⋯O inter­action and as an acceptor in two N—H⋯O inter­actions. The propano­ylurea mol­ecules inter­act with each other *via* N—H⋯O hydrogen bonds. C—H⋯O inter­actions also stabilize the crystal structure.

## Related literature

For the preparation of heterocyclic compounds, see: Weinberg & Tilak (1982 ▶); Katritzky & Lagowski (1971 ▶); Sicker *et al.* (1995 ▶). For bond lengths and angles in similar compounds, see: Cai *et al.* (2011 ▶); Yakimanski *et al.* (1997 ▶).
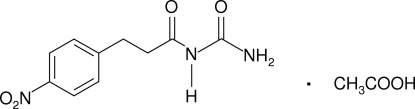


## Experimental

### 

#### Crystal data

C_10_H_11_N_3_O_4_·C_2_H_4_O_2_
*M* *_r_* = 297.27
Triclinic, 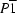

*a* = 7.4252 (3) Å
*b* = 7.9601 (3) Å
*c* = 11.4375 (4) Å
α = 92.736 (3)°
β = 92.939 (3)°
γ = 91.091 (3)°
*V* = 674.20 (4) Å^3^
*Z* = 2
Mo *K*α radiation
μ = 0.12 mm^−1^
*T* = 100 K
0.40 × 0.20 × 0.10 mm

#### Data collection

Oxford Diffraction SuperNova diffractometer
Absorption correction: integration (*ABSORB*; DeTitta, 1985 ▶) *T*_min_ = 0.954, *T*_max_ = 0.988
16680 measured reflections
3913 independent reflections
3563 reflections with *I* > 2σ(*I*)
*R*_int_ = 0.026

#### Refinement

*R*[*F* ^2^ > 2σ(*F* ^2^)] = 0.041
*wR*(*F* ^2^) = 0.111
*S* = 1.05
3913 reflections
250 parameters
All H-atom parameters refined
Δρ_max_ = 0.41 e Å^−3^
Δρ_min_ = −0.32 e Å^−3^

### 

Data collection: *CrysAlis CCD* (Oxford Diffraction, 2009 ▶); cell refinement: *CrysAlis RED* (Oxford Diffraction, 2009 ▶); data reduction: *CrysAlis RED*; program(s) used to solve structure: *SHELXS97* (Sheldrick, 2008 ▶); program(s) used to refine structure: *SHELXL97* (Sheldrick, 2008 ▶); molecular graphics: *ORTEPIII* (Burnett & Johnson, 1996 ▶) and *ORTEP-3 for Windows* (Farrugia, 1997 ▶); software used to prepare material for publication: *enCIFer* (Allen *et al.*, 2004 ▶).

## Supplementary Material

Crystal structure: contains datablock(s) global, I. DOI: 10.1107/S1600536811039729/fy2022sup1.cif

Structure factors: contains datablock(s) I. DOI: 10.1107/S1600536811039729/fy2022Isup2.hkl

Supplementary material file. DOI: 10.1107/S1600536811039729/fy2022Isup3.cml

Additional supplementary materials:  crystallographic information; 3D view; checkCIF report

## Figures and Tables

**Table 1:** Hydrogen-bond geometry (Å, °)

*D*—H⋯*A*	*D*—H	H⋯*A*	*D*⋯*A*	*D*—H⋯*A*
O6—H⋯O4^i^	0.90 (2)	1.76 (2)	2.6492 (12)	166 (2)
N2—H9⋯O4^ii^	0.863 (15)	1.998 (15)	2.8611 (12)	178.7 (15)
N3—H10⋯O3	0.884 (15)	2.036 (16)	2.6892 (12)	129.8 (13)
N3—H10⋯O5^iii^	0.884 (15)	2.349 (15)	2.9434 (13)	124.7 (13)
N3—H11⋯O5^iv^	0.899 (16)	2.017 (16)	2.9050 (12)	169.1 (14)
C2—H2⋯O3^i^	0.980 (16)	2.550 (17)	3.3434 (14)	138.0 (13)
C3—H3⋯O5^v^	0.957 (16)	2.536 (15)	3.4793 (13)	168.6 (12)
C5—H5⋯O2^iv^	0.945 (16)	2.463 (16)	3.3724 (14)	161.6 (12)
C8—H81⋯O1^vi^	0.950 (15)	2.496 (15)	3.4266 (15)	166.3 (13)

Symmetry codes: (i) 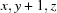; (ii) 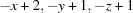; (iii) 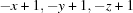; (iv) 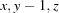; (v) 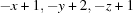; (vi) 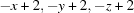.

**Fig. 1.**
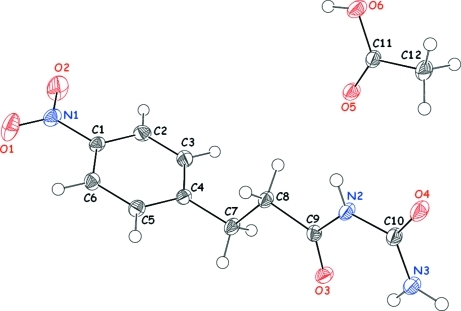
ORTEP-3 drawing of the title compound with the atom-numbering scheme. Ellispoids are drawn at the 50 % probability level.

**Fig. 2.**
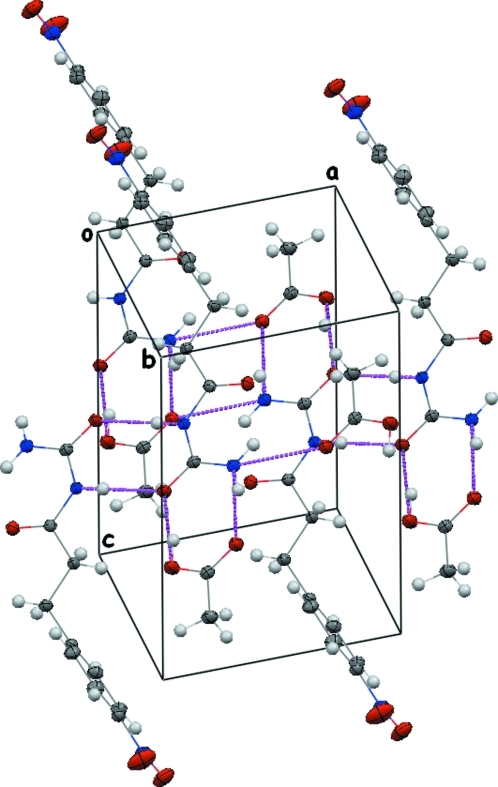
Molecular packing and hydrogen bond pattern.

## supplementary crystallographic information

### Comment

Over the past few years, significant research has been directed toward the development of new technologies for environment-friendly processes, such as electrochemical synthesis and green chemistry, which are both economically and technologically feasible. Electrochemistry seems to be a method of choice to prepare various heterocyclic compounds, because the anodic oxidation and the cathodic reduction allow the selective preparation of the active intermediates (Weinberg & Tilak, 1982). However, obtaining aniline products is generally not easy. The chemical reductions are not always selective and often lead to mixtures (Katritzky & Lagowski, 1971). In particular, the reduction of substituted nitrobenzenes with controlled potential can produce a number of heterocycles (Sicker *et al.*, 1995).

The present paper reports the crystal structure determination and analysis of a new organic compound. The asymmetric unit contains one 1-(3-(4-nitrophenyl)propanoyl)urea and one acetic acid molecule (Fig. 1). The cohesion and stability of the crystal is provided by N—H···O, O—H···O and C—H···O hydrogen bonds (Table 1).

These interactions form molecular tapes composed of alternating acetic acid and 1-(3-(4-nitrophenyl)propanoyl)urea molecules (Fig. 2).

All bond lengths and angles are within usual values and are comparable to those observed in similar compounds (Cai *et al.*, 2011; Yakimanski *et al.*, 1997).

### Experimental

The electrochemical studies were carried out in ethanol using sodium acetate buffer (1 N) as electrolyte support. We worked on a potential of -1 V/SCE with mercury electrode. The electroreduction of ethyl 3-(4-nitrophenyl)propanoate gave a mixture of the title compound and 6-hydroxy-2,3-dihydroinden-1-one. Only the title compound is soluble in ethanol, which allowed easy separation of the two reaction products. The title compound was recrystallized from ethanol/acetic acid (1:1), and gave crystals that melt at 110°C. 6-Hydroxy-2,3-dihydroinden-1-one, after recrystallization from ethanol/water (2:1), was found to melt at 156°C.

### Refinement

The electron density of the H atoms was clearly identified in the Fourier difference map, and the atomic coordinates and isotropic displacements parameters of the H atoms were refined freely.

### Crystal data

**Table:**

C_10_H_11_N_3_O_4_·C_2_H_4_O_2_	*Z* = 2
*M**_r_* = 297.27	*F*(000) = 312
Triclinic, *P*1	*D*_x_ = 1.464 Mg m^−^^3^
*a* = 7.4252 (3) Å	Mo *K*α radiation, λ = 0.71073 Å
*b* = 7.9601 (3) Å	Cell parameters from 4625 reflections
*c* = 11.4375 (4) Å	θ = 3.2–30.0°
α = 92.736 (3)°	µ = 0.12 mm^−^^1^
β = 92.939 (3)°	*T* = 100 K
γ = 91.091 (3)°	Prism, yellow
*V* = 674.20 (4) Å^3^	0.40 × 0.20 × 0.10 mm

### Data collection

**Table:**

Oxford Diffraction SuperNova diffractometer	3913 independent reflections
Radiation source: fine-focus sealed tube	3563 reflections with *I* > 2σ(*I*)
graphite	*R*_int_ = 0.026
ω scans	θ_max_ = 30.0°, θ_min_ = 3.2°
Absorption correction: integration (*ABSORB*; DeTitta, 1985)	*h* = −10→10
*T*_min_ = 0.954, *T*_max_ = 0.988	*k* = −11→11
16680 measured reflections	*l* = 0→16

### Refinement

**Table:**

Refinement on *F*^2^	Primary atom site location: structure-invariant direct methods
Least-squares matrix: full	Secondary atom site location: difference Fourier map
*R*[*F*^2^ > 2σ(*F*^2^)] = 0.041	Hydrogen site location: inferred from neighbouring sites
*wR*(*F*^2^) = 0.111	All H-atom parameters refined
*S* = 1.05	*w* = 1/[σ^2^(*F*_o_^2^) + (0.0556*P*)^2^ + 0.2344*P*] where *P* = (*F*_o_^2^ + 2*F*_c_^2^)/3
3913 reflections	(Δ/σ)_max_ < 0.001
250 parameters	Δρ_max_ = 0.41 e Å^−^^3^
0 restraints	Δρ_min_ = −0.32 e Å^−^^3^

### Special details

**Table:**

Geometry. All s.u.'s (except the s.u. in the dihedral angle between two l.s. planes) are estimated using the full covariance matrix. The cell s.u.'s are taken into account individually in the estimation of s.u.'s in distances, angles and torsion angles; correlations between s.u.'s in cell parameters are only used when they are defined by crystal symmetry. An approximate (isotropic) treatment of cell s.u.'s is used for estimating s.u.'s involving l.s. planes.
Refinement. Refinement of *F*^2^ against ALL reflections. The weighted *R*-factor *wR* and goodness of fit *S* are based on *F*^2^, conventional *R*-factors *R* are based on *F*, with *F* set to zero for negative *F*^2^. The threshold expression of *F*^2^ > 2σ(*F*^2^) is used only for calculating *R*-factors(gt) *etc*. and is not relevant to the choice of reflections for refinement. *R*-factors based on *F*^2^ are statistically about twice as large as those based on *F*, and *R*- factors based on ALL data will be even larger.

### Fractional atomic coordinates and isotropic or equivalent isotropic displacement parameters (Å^2^)

**Table:**

	*x*	*y*	*z*	*U*_iso_*/*U*_eq_	
N2	0.77505 (12)	0.49422 (11)	0.56957 (8)	0.01868 (18)	
O3	0.51190 (10)	0.47679 (9)	0.66343 (7)	0.02065 (17)	
C9	0.64971 (13)	0.55423 (12)	0.64495 (8)	0.01614 (18)	
C10	0.77604 (14)	0.33654 (13)	0.51251 (9)	0.01820 (19)	
O4	0.91036 (11)	0.30011 (10)	0.45541 (7)	0.02409 (18)	
N3	0.63746 (13)	0.23181 (11)	0.52143 (8)	0.01982 (18)	
O5	0.71569 (11)	0.90282 (10)	0.41754 (7)	0.02333 (17)	
O6	0.95760 (12)	1.01068 (11)	0.33799 (8)	0.0291 (2)	
C11	0.84078 (14)	0.88799 (13)	0.35312 (9)	0.0189 (2)	
C12	0.87300 (17)	0.73163 (14)	0.28067 (10)	0.0242 (2)	
H71	0.450 (2)	0.7953 (19)	0.7578 (13)	0.025 (4)*	
H81	0.820 (2)	0.714 (2)	0.7386 (14)	0.029 (4)*	
H6	0.865 (2)	1.0680 (19)	1.1039 (14)	0.028 (4)*	
H2	0.619 (2)	1.350 (2)	0.8551 (15)	0.038 (4)*	
H82	0.710 (2)	0.8064 (19)	0.6429 (13)	0.026 (4)*	
H5	0.763 (2)	0.822 (2)	0.9966 (13)	0.029 (4)*	
H72	0.556 (2)	0.687 (2)	0.8510 (14)	0.029 (4)*	
H3	0.524 (2)	1.101 (2)	0.7488 (14)	0.029 (4)*	
H9	0.871 (2)	0.555 (2)	0.5624 (14)	0.030 (4)*	
H10	0.545 (2)	0.263 (2)	0.5625 (14)	0.029 (4)*	
H11	0.646 (2)	0.127 (2)	0.4896 (14)	0.033 (4)*	
H121	0.832 (2)	0.635 (2)	0.3199 (16)	0.041 (5)*	
H	0.927 (3)	1.101 (3)	0.383 (2)	0.064 (6)*	
H122	0.999 (3)	0.723 (2)	0.2602 (16)	0.047 (5)*	
H123	0.802 (3)	0.738 (3)	0.207 (2)	0.067 (6)*	
C4	0.63442 (13)	0.93684 (12)	0.86240 (9)	0.01726 (19)	
C1	0.75280 (14)	1.22675 (12)	0.98721 (9)	0.0184 (2)	
C8	0.70192 (14)	0.72292 (12)	0.70362 (9)	0.01779 (19)	
N1	0.81408 (14)	1.38040 (12)	1.05424 (9)	0.0242 (2)	
C5	0.73579 (15)	0.92833 (13)	0.96774 (9)	0.0193 (2)	
C7	0.56967 (15)	0.77809 (13)	0.79493 (9)	0.0203 (2)	
C3	0.59529 (15)	1.09439 (14)	0.82046 (9)	0.0218 (2)	
O1	0.90310 (14)	1.36682 (12)	1.14622 (9)	0.0386 (2)	
C2	0.65298 (16)	1.24075 (13)	0.88280 (10)	0.0221 (2)	
C6	0.79598 (15)	1.07320 (13)	1.03125 (9)	0.0195 (2)	
O2	0.77247 (18)	1.51546 (11)	1.01607 (10)	0.0464 (3)	

### Atomic displacement parameters (Å^2^)

**Table:**

	*U*^11^	*U*^22^	*U*^33^	*U*^12^	*U*^13^	*U*^23^
N2	0.0173 (4)	0.0162 (4)	0.0221 (4)	−0.0037 (3)	0.0049 (3)	−0.0059 (3)
O3	0.0193 (4)	0.0183 (3)	0.0243 (4)	−0.0037 (3)	0.0052 (3)	−0.0026 (3)
C9	0.0170 (4)	0.0159 (4)	0.0154 (4)	0.0010 (3)	0.0007 (3)	−0.0014 (3)
C10	0.0188 (5)	0.0172 (4)	0.0182 (4)	−0.0014 (3)	0.0017 (3)	−0.0035 (3)
O4	0.0215 (4)	0.0205 (4)	0.0298 (4)	−0.0053 (3)	0.0099 (3)	−0.0104 (3)
N3	0.0193 (4)	0.0173 (4)	0.0227 (4)	−0.0028 (3)	0.0052 (3)	−0.0036 (3)
O5	0.0230 (4)	0.0203 (4)	0.0266 (4)	−0.0034 (3)	0.0065 (3)	−0.0053 (3)
O6	0.0293 (4)	0.0218 (4)	0.0359 (5)	−0.0090 (3)	0.0145 (4)	−0.0114 (3)
C11	0.0194 (5)	0.0183 (4)	0.0186 (4)	−0.0016 (4)	0.0001 (4)	−0.0027 (3)
C12	0.0280 (6)	0.0189 (5)	0.0252 (5)	−0.0010 (4)	0.0050 (4)	−0.0064 (4)
C4	0.0173 (4)	0.0162 (4)	0.0183 (4)	−0.0010 (3)	0.0063 (3)	−0.0033 (3)
C1	0.0206 (5)	0.0138 (4)	0.0209 (5)	−0.0014 (3)	0.0050 (4)	−0.0031 (3)
C8	0.0171 (4)	0.0164 (4)	0.0195 (4)	−0.0024 (3)	0.0036 (3)	−0.0045 (3)
N1	0.0284 (5)	0.0159 (4)	0.0279 (5)	−0.0018 (3)	0.0036 (4)	−0.0043 (3)
C5	0.0236 (5)	0.0135 (4)	0.0208 (5)	0.0018 (4)	0.0032 (4)	−0.0012 (3)
C7	0.0197 (5)	0.0189 (5)	0.0219 (5)	−0.0034 (4)	0.0062 (4)	−0.0068 (4)
C3	0.0247 (5)	0.0215 (5)	0.0192 (5)	−0.0001 (4)	0.0003 (4)	0.0012 (4)
O1	0.0432 (6)	0.0270 (5)	0.0426 (5)	0.0031 (4)	−0.0148 (4)	−0.0129 (4)
C2	0.0282 (5)	0.0160 (4)	0.0225 (5)	0.0010 (4)	0.0028 (4)	0.0029 (4)
C6	0.0228 (5)	0.0174 (4)	0.0180 (4)	0.0016 (4)	0.0015 (4)	−0.0020 (3)
O2	0.0810 (8)	0.0129 (4)	0.0435 (6)	−0.0018 (4)	−0.0115 (5)	−0.0004 (4)

### Geometric parameters (Å, °)

**Table:**

N2—C9	1.3776 (12)	C4—C7	1.5064 (14)
N2—C10	1.3871 (12)	C1—C6	1.3811 (14)
N2—H9	0.866 (17)	C1—C2	1.3831 (15)
O3—C9	1.2175 (13)	C1—N1	1.4643 (13)
C9—C8	1.5070 (13)	C8—C7	1.5250 (14)
C10—O4	1.2510 (12)	C8—H81	0.950 (16)
C10—N3	1.3230 (14)	C8—H82	0.987 (15)
N3—H10	0.884 (17)	N1—O2	1.2187 (13)
N3—H11	0.898 (18)	N1—O1	1.2237 (14)
O5—C11	1.2189 (13)	C5—C6	1.3872 (14)
O6—C11	1.3174 (13)	C5—H5	0.942 (16)
O6—H	0.90 (2)	C7—H71	0.981 (15)
C11—C12	1.4920 (14)	C7—H72	1.000 (16)
C12—H121	0.960 (18)	C3—C2	1.3854 (15)
C12—H122	0.98 (2)	C3—H3	0.956 (15)
C12—H123	0.97 (2)	C2—H2	0.977 (17)
C4—C5	1.3926 (15)	C6—H6	0.958 (16)
C4—C3	1.3944 (14)		
			
C9—N2—C10	127.24 (9)	C9—C8—C7	112.08 (8)
C9—N2—H9	117.7 (11)	C9—C8—H81	107.3 (10)
C10—N2—H9	114.6 (11)	C7—C8—H81	110.8 (10)
O3—C9—N2	123.04 (9)	C9—C8—H82	108.7 (9)
O3—C9—C8	123.57 (9)	C7—C8—H82	110.8 (9)
N2—C9—C8	113.38 (8)	H81—C8—H82	106.8 (13)
O4—C10—N3	123.15 (9)	O2—N1—O1	123.29 (10)
O4—C10—N2	117.71 (9)	O2—N1—C1	118.30 (10)
N3—C10—N2	119.13 (9)	O1—N1—C1	118.41 (9)
C10—N3—H10	120.3 (11)	C6—C5—C4	121.10 (9)
C10—N3—H11	117.3 (11)	C6—C5—H5	119.4 (10)
H10—N3—H11	122.2 (15)	C4—C5—H5	119.4 (10)
C11—O6—H	107.8 (14)	C4—C7—C8	111.51 (8)
O5—C11—O6	123.10 (10)	C4—C7—H71	109.6 (9)
O5—C11—C12	123.45 (10)	C8—C7—H71	110.7 (9)
O6—C11—C12	113.42 (9)	C4—C7—H72	108.9 (9)
C11—C12—H121	109.7 (11)	C8—C7—H72	109.1 (9)
C11—C12—H122	112.0 (11)	H71—C7—H72	106.8 (13)
H121—C12—H122	112.5 (15)	C2—C3—C4	121.03 (10)
C11—C12—H123	107.4 (13)	C2—C3—H3	119.5 (10)
H121—C12—H123	108.7 (17)	C4—C3—H3	119.4 (10)
H122—C12—H123	106.3 (17)	C1—C2—C3	118.28 (10)
C5—C4—C3	118.86 (9)	C1—C2—H2	121.2 (10)
C5—C4—C7	120.31 (9)	C3—C2—H2	120.5 (10)
C3—C4—C7	120.83 (10)	C1—C6—C5	118.21 (10)
C6—C1—C2	122.52 (9)	C1—C6—H6	120.4 (9)
C6—C1—N1	118.62 (9)	C5—C6—H6	121.4 (9)
C2—C1—N1	118.86 (9)		
			
C10—N2—C9—O3	−6.33 (17)	C5—C4—C7—C8	−92.93 (12)
C10—N2—C9—C8	172.56 (9)	C3—C4—C7—C8	86.63 (12)
C9—N2—C10—O4	−173.81 (10)	C9—C8—C7—C4	173.53 (9)
C9—N2—C10—N3	5.63 (16)	C5—C4—C3—C2	−1.10 (16)
O3—C9—C8—C7	4.31 (14)	C7—C4—C3—C2	179.33 (10)
N2—C9—C8—C7	−174.57 (9)	C6—C1—C2—C3	−0.24 (17)
C6—C1—N1—O2	−178.56 (11)	N1—C1—C2—C3	−179.77 (10)
C2—C1—N1—O2	1.00 (16)	C4—C3—C2—C1	0.88 (17)
C6—C1—N1—O1	0.81 (15)	C2—C1—C6—C5	−0.18 (16)
C2—C1—N1—O1	−179.63 (11)	N1—C1—C6—C5	179.36 (9)
C3—C4—C5—C6	0.67 (15)	C4—C5—C6—C1	−0.04 (16)
C7—C4—C5—C6	−179.76 (9)		

### Hydrogen-bond geometry (Å, °)

**Table:**

*D*—H···*A*	*D*—H	H···*A*	*D*···*A*	*D*—H···*A*
O6—H···O4^i^	0.90 (2)	1.76 (2)	2.6492 (12)	166 (2)
N2—H9···O4^ii^	0.863 (15)	1.998 (15)	2.8611 (12)	178.7 (15)
N3—H10···O3	0.884 (15)	2.036 (16)	2.6892 (12)	129.8 (13)
N3—H10···O5^iii^	0.884 (15)	2.349 (15)	2.9434 (13)	124.7 (13)
N3—H11···O5^iv^	0.899 (16)	2.017 (16)	2.9050 (12)	169.1 (14)
C2—H2···O3^i^	0.980 (16)	2.550 (17)	3.3434 (14)	138.0 (13)
C3—H3···O5^v^	0.957 (16)	2.536 (15)	3.4793 (13)	168.6 (12)
C5—H5···O2^iv^	0.945 (16)	2.463 (16)	3.3724 (14)	161.6 (12)
C8—H81···O1^vi^	0.950 (15)	2.496 (15)	3.4266 (15)	166.3 (13)

Symmetry codes: (i) *x*, *y*+1, *z*; (ii) −*x*+2, −*y*+1, −*z*+1;  (iii) −*x*+1, −*y*+1, −*z*+1; (iv) *x*, *y*−1, *z*; (v) −*x*+1, −*y*+2, −*z*+1; (vi) −*x*+2, −*y*+2, −*z*+2.

## References

[bb1] Allen, F. H., Johnson, O., Shields, G. P., Smith, B. R. & Towler, M. (2004). *J. Appl. Cryst.* **37**, 335–338.

[bb2] Burnett, M. N. & Johnson, C. K. (1996). *ORTEPIII* Report ORNL-6895. Oak Ridge National Laboratory, Tennessee, USA.

[bb3] Cai, Q., Fei, Z. & Li, L. (2011). *Acta Cryst.* E**67**, o1494. 10.1107/S1600536811018757 PMC3120399 21754862

[bb4] DeTitta, G. T. (1985). *J. Appl. Cryst.* **18**, 75–79.

[bb5] Farrugia, L. J. (1997). *J. Appl. Cryst.* **30**, 565.

[bb6] Katritzky, A. R. & Lagowski, J. M. (1971). In *Chemistry of the Heterocyclic N-oxides* London: Academic Press.

[bb7] Oxford Diffraction (2009). *CrysAlis CCD* and *CrysAlis RED* Oxford Diffraction Ltd, Yarnton, England.

[bb8] Sheldrick, G. M. (2008). *Acta Cryst.* A**64**, 112–122. 10.1107/S0108767307043930 18156677

[bb9] Sicker, D., Hartenstein, H., Mouats, C., Hazards, R. & Tallec, A. (1995). *Electrochim. Acta*, **40**, 1669–1674.

[bb10] Weinberg, N. L. & Tilak, B. V. (1982). *Technique of Electroorganic Synthesis, Scale-up and Engineering Aspects*, Vol. 5. New York: Wiley.

[bb11] Yakimanski, A. V., Kolb, U., Matveeva, G. N., Voigt-Martin, I. G. & Tenkovtsev, A. V. (1997). *Acta Cryst.* A**53**, 603–614.

